# Associations Between MicroRNA and Abdominal Aortic Aneurysm Diameter Differ by Sex

**DOI:** 10.3390/biomedicines14030507

**Published:** 2026-02-25

**Authors:** Jonas Wallinder, Anne Kunath, Dick Wågsäter, Martin Björck, Anders Wanhainen

**Affiliations:** 1 Department of Surgical Sciences, Section of Vascular Surgery, Uppsala University, 751 85 Uppsala, Sweden; martin.bjorck@uu.se (M.B.); anders.wanhainen@uu.se (A.W.); 2 Department of Surgery, Sundsvall District Hospital, 856 43 Sundsvall, Sweden; 3 Department of Medical Cell Biology, Uppsala University, 751 23 Uppsala, Swedendick.wagsater@mcb.uu.se (D.W.); 4 Institute of Clinical Medicine, University of Tartu, 50406 Tartu, Estonia; 5 Department of Surgical and Perioperative Sciences, Umeå University, 901 85 Umeå, Sweden

**Keywords:** aortic aneurysm, abdominal, sex, microRNAs, women

## Abstract

**Objective:** Abdominal aortic aneurysm (AAA) epidemiology differs significantly between the sexes; the biological factors behind this are mostly unknown. MicroRNAs (miRNAs) are short RNA molecules providing post-transcriptional regulation of protein synthesis. Several miRNAs have been associated with the development and growth of AAA, but only in men. We investigated whether the associations between some selected miRNAs and aortic size differ by sex and the possible target pathways for such differences. **Methods:** A cross-sectional study included subjects with AAA (30–58 mm) and normal aortas. Clinical data were collected through questionnaires. Abdominal aortic diameters were measured using ultrasound. The levels of 17 miRNAs were measured in plasma. The association between miRNA levels, aortic diameter, and sex were analysed using multivariable linear regression. **Results:** A total of 242 subjects were included, with 85 women and 157 men. In the group with aortic diameters below 30 mm were 122 men (15–29 mm) and 50 women (13–29 mm). There were 35 men (30–54 mm) and 35 women (30–58 mm) with AAA. The associations between six miRNAs and aortic diameter were influenced by sex: miR-125 (*p* = 0.013), miR-128–1 (*p* = 0.017), miR-24 (*p* = 0.013), miR-26a (*p* = 0.022), miR-93 (*p* = 0.0015), and miR-194 (*p* = 0.013). Bioinformatic analysis indicated Hippo and TGF-beta as the two signalling pathways most likely affected by these differences. **Conclusions:** This exploratory study found sex differences in the associations between miRNA levels and aortic diameter, involving signalling pathways that control organ size and maintain tissue homeostasis by regulating cell proliferation, survival, and differentiation.

## 1. Introduction

Abdominal aortic aneurysm (AAA) is about 3–5 times more common among men than among women [1,2,3], but when affected, women suffer a worse prognosis [4,5,6,7,8,9,10]. These significant sex-dependent differences in AAA epidemiology have not been fully explained [11]. Sex differences in risk factor exposure and body size are contributing factors, while the importance of sex hormones is debated [12,13,14,15,16].

MicroRNAs (miRNAs) are short non-coding RNA molecules with 18–25 bases arranged in a “hairpin” configuration. miRNAs are active in the regulation of protein synthesis, primarily through inhibiting messenger-RNA (not to be confused with miRNA) translation or marking messenger-RNA for rapid degradation, thereby reducing protein synthesis from the messenger-RNA [17]. When miRNAs are secreted from the cell, donor cells may influence gene expression in receiver cells in a hormone-like manner, as circulating miRNA can be taken up from plasma and modulate intracellular protein synthesis, in some cases promoting aneurysm development [18].

The role of miRNAs in secondary regulation or fine-tuning of protein synthesis and their hormone-like function when secreted suggest that miRNA levels can be viewed as a function of the current molecular biological state of an organism. Circulating miRNA profiles have been associated with diseases and disease stages, including AAA [19,20,21]. However, these studies have mainly been conducted on men, and sex differences are poorly studied.

The aim of the current study is to explore associations between aortic diameter and the levels of some selected miRNAs and whether these associations are different between the sexes.

## 2. Materials and Methods

Men and women under surveillance for small AAAs as well as those with normal aortas at two centres were asked to participate. Men with normal aortas were recruited as part of routine population-based AAA screening, and women were recruited as part of a population-based screening study [2]. All participants underwent an ultrasound measurement of their aorta within 90 days of when their blood samples were collected, and the participants then completed a questionnaire on medical and family history, smoking, and current medication. Blood samples were centrifuged at 2000× *g* for 10 min at room temperature, and plasma was frozen at −70 °C.

A total of seventeen miRNAs were analysed; eight miRNAs were selected based on our previous findings of miRNAs associated with AAA in a male population [21], and nine additional miRNAs were selected based on a literature review. Total RNA was extracted from plasma using the miRCURY™ RNA isolation kit—biofluids (Exiqon, Vedbaek, Denmark) [22], as described in more depth in a previous study [19]. In brief, plasma was mixed with Lysis Solution BF containing one µg carrier-RNA per 60 µL Lysis Solution BF. UniSp6 was added as the control for the reverse transcription step. Controls (negative and RNA spike-in) indicated the good technical performance of the profiling experiment. Then, 7 µL RNA was reverse-transcribed in a 35 µL reaction volume using the miRCURY LNA™ Universal RT microRNA PCR, Polyadenylation and cDNA synthesis kit (Exiqon). Each RNA sample was successfully polyadenylated and reverse-transcribed into cDNA. cDNA was diluted 50 times and assayed in 10 µL PCR reactions, according to the protocol for miRCURY LNA™ Universal RT microRNA PCR, using ExiLENT SYBR^®^ Green master mix. The plasma panel included 17 miRs validated as being expressed in plasma, based on our previous experience, with 752 different miRs screened in plasma and validation performed by Exiqon [16]. Negative controls, excluding template from the reverse transcription reaction, were performed and profiled like the samples. Amplification was performed in a 7900 Fast Real-time PCR Sequence Detector (Applied Biosystems; Thermo Fisher Scientific, Inc., Waltham, MA, USA), and samples were quantified using a standard curve. The amplification efficiency was calculated using algorithms similar to the LinReg software version 11.0. All assays were inspected for distinct melting curves, and the Tm was checked to be within known specifications for the assay. Furthermore, assays must be detected with 5 Cqs less than the negative control and with Cq < 37 to be included in the data analysis. Cq was calculated as the second derivative.

Continuous variables are presented as medians with interquartile ranges; binary and categorical variables are presented as the number of cases and percentage. Ahead of regression analysis, miRNA levels and aortic diameters were standardised to a mean of 0 with a standard deviation of 1. For presentation, aortic diameters were transformed back to original values, and miRNA levels were presented as deviations from the mean in standard deviations. Associations between miRNA levels and aortic diameter were calculated using linear regressions with miRNA level as the dependent variable and aortic diameter, sex, the interaction between sex and aortic diameter, smoking status, and the other covariates in Table 1 as independent variables. The interaction term was introduced to assess how sex modifies the association between aortic diameter and miRNA level.

Each miRNA level was analysed using a separate regression. *p*-values were adjusted for multiple testing according to Holm and considered significant below 0.05. Only estimates of associations of miRNA levels with aortic diameter, sex, and their interaction were considered for this study. A power calculation using InteractionPowerR calculated a power of 85%, using expected main and interaction correlations of −0.2 and a 10% diameter measurement error [23].

The web-based mirPath software version 3.0 [24] evaluated the likely effects of differences in miRNA levels. The software associates groups of miRNAs with pathways in the KEGG (Kyoto Encyclopedia of Genes and Genomes) database of molecular interactions [25] by implementing statistical and bioinformatics techniques.

## 3. Results

**Figure 1:**
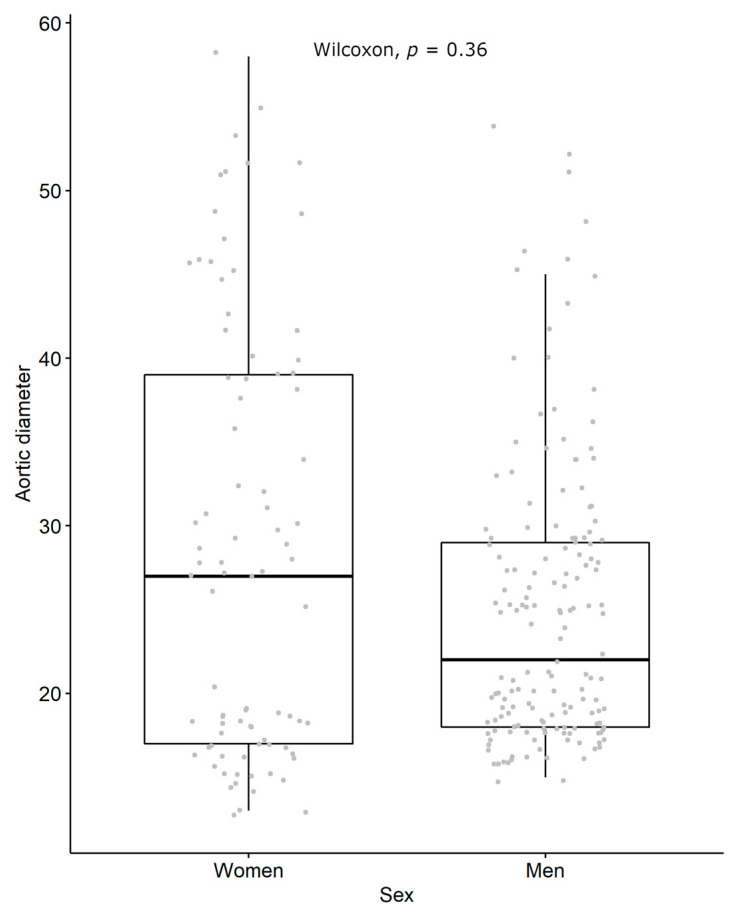
Distribution of aortic diameters. A total of 242 participants, with 85 women and 157 men.

Among the 242 subjects included, 85 were women and 157 men; their clinical characteristics are described in Table 1. Chronic obstructive pulmonary disease was more common among the women (15.3% vs. 6.4%, *p*: 0.021), and there were more active smokers among the women (26% vs. 11%, *p*: 0.006). There was a slight difference in the distribution of aortic diameters between the men and women (Figure 1).

**Figure 2:**
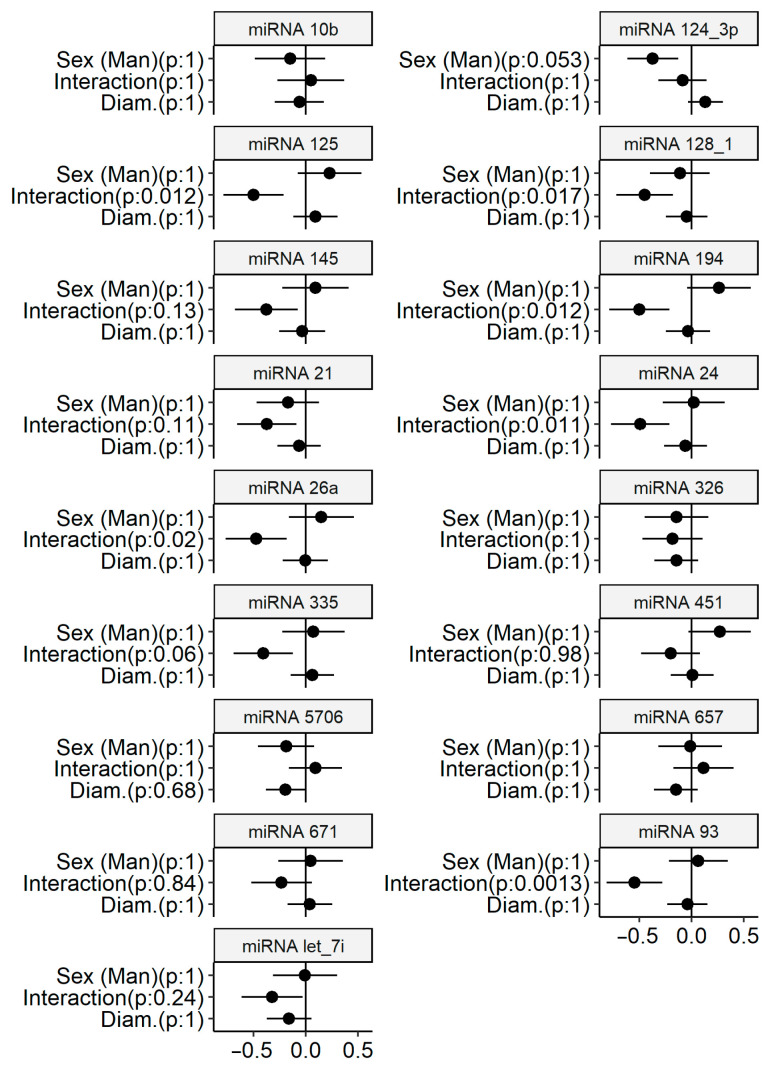
Point estimates of linear regressions with 95% confidence intervals and adjusted *p*-values. There were 242 participants, with 85 women and 157 men.

**Figure 3:**
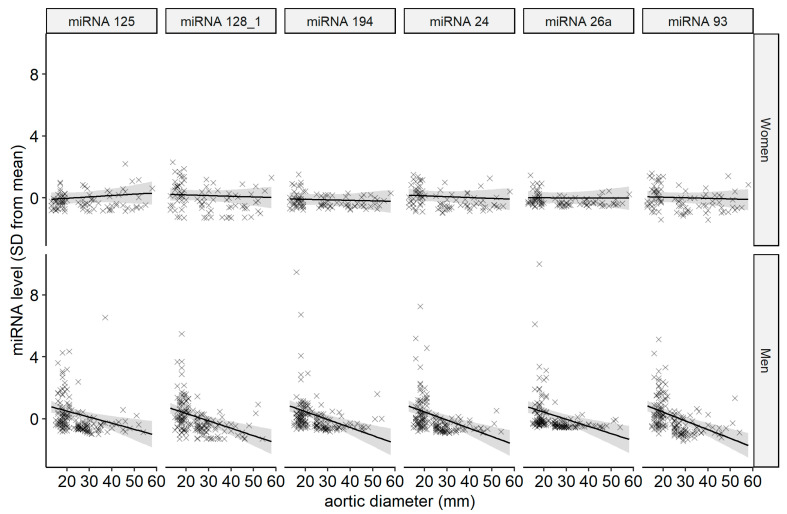
miRNA and aortic diameter, with unadjusted regression lines with 95% confidence intervals. There were 242 participants, with 85 women and 157 men.

Linear regression was performed for each miRNA, estimating associations with aortic diameter, sex, and their interaction using other clinical variables as covariates (Table 2). The complete table of estimates from the regressions is available as an e-supplement (Table S1). Aortic diameter was treated as a continuous variable to reduce effects of measurement errors and avoid loss of statistical power, as categorization may also introduce misclassification [26]. Regression estimates are also presented as a figure, where each pane represents a regression (Figure 2). In the regressions of six miRNAs (miR-125, miR-128-1, miR-24, miR-26a, miR-93, and miR-94), there was a significant interaction between sex and the association between aortic diameter and miRNA level. All the significant interaction estimates indicate that miRNAs were downregulated with larger aortic diameter among the men but not among the women (Figure 3).

Each miRNA was analysed using a separate regression; the covariates in Table 1 are included as confounders. The *p*-values were adjusted according to Holm; the aortic diameter and miRNA levels are standardised to a mean of 0 and standard deviation of 1. There were 242 participants, with 85 women and 157 men.

The levels of miR-124-3p were associated with sex (−0.37, −0.61–−0.13; *p*: 0.0031), but the association did not hold up when adjusted for multiple testing (adj. *p*: 0.052). MiR-5706 levels were associated with aortic diameter (−0.2, −0.38–−0.009; *p*: 0.04), but this association did not remain after adjustment for multiple testing (adj. *p*: 0.68).

An analysis using mirPath presented the Hippo signalling pathway as having the strongest association with the six significant miRNAs, and the associated TGF-beta signalling pathway was shown to be the third most likely target (Table 3).

## 4. Discussion

This exploratory study found an association between some selected miRNAs and aortic diameters, but interestingly, the association was strongly sex-dependent. The interaction between sex and aortic diameter indicates that the association is only present in men.

miRNA has previously been associated with AAA development; these studies have, however, been conducted solely on men or predominantly on men. A thorough study by Maegdefessel [27], using tissue and plasma from both rodents and humans, indicated miR-24 regulation of vascular inflammation as an important factor in AAA development. However, the subjects were male-only. The sex differences identified in this study indicate the importance of considering sex in future pathophysiological studies of AAA disease and that results from all-male studies cannot necessarily be extrapolated to women.

Differences between the sexes in miRNA expression have been linked to other diseases, predominantly cancer and stroke, but have not previously been shown for AAA. MiRNA levels have been associated with oestrogen levels [28] and vascular ageing, and specifically the inflammatory ageing process [29,30]. Inflammatory vascular ageing is closely related to processes identified in AAA development, such as migration and activation of inflammatory cells and degeneration of the extracellular matrix. However, none of the miRNAs with significant sex interactions in this study have been associated with oestrogen regulation [31].

The biological implications of the observed sex difference in the association between miRNAs and aortic size are challenging to assess from this limited study. Causality cannot be determined from the present study; although altered miRNA levels may contribute to aortic remodelling and subsequent dilatation, they may also represent a reactive response to changes in the aneurysmal vessel wall.

However, available bioinformatics tools can provide guidance on the potential effects of altered miRNA expression. MirPath offers a probabilistic association between altered miRNA levels and signalling pathways. The pathway with the most significant association was the Hippo signalling pathway, associated with vascular remodelling and vascular disease [32]; its increased activity reduces cell proliferation and causes vascular smooth muscle cell apoptosis [33], a well-established aspect of AAA development. Oestrogen (17β-estradiol) stimulates the Hippo signalling pathway through a plasma membrane receptor (GPER/GPR30) in certain cell types [34].

The syndromic aortic aneurysm diseases Marfan syndrome and Loeys–Dietz syndrome are caused by mutations in genes affecting TGF-beta signalling [35]. TGF-beta, through TGF-beta receptors, activates SMAD2 and SMAD3, which enter the nucleus to regulate the expression of target genes [36]. TGF-beta activation can also influence levels of miRNAs associated with AAA [37]. The Hippo signalling pathway activates YAP/TAZ, which regulates SMAD accumulation in the nucleus [38]. As a result of these mechanisms, there is extensive cross-talk between the TGF-beta and Hippo signalling pathways [39,40], suggesting an association between the Hippo signalling pathway and aneurysm formation. The association between the significant miRNAs in this study and both Hippo signalling and TGF-beta signalling strengthen these findings.

The results of this study suggest that among men with larger aortas, there is a difference in miRNA levels associated with a lower suppression of the Hippo signalling pathway. However, among women, the same pattern of low suppression is present regardless of aortic diameter. This result is somewhat perplexing. Since AAA is more common among men, we expected to find women with AAA more aligned to a male miRNA pattern rather than the opposite.

These changes should be interpreted as part of a complex cellular response. As this is not a longitudinal study, it cannot be excluded that low levels of the examined miRNAs in men reflect a biological state predisposing to AAA development, whereas women may exhibit alternative protective mechanisms.

A possible explanation is that oestrogen’s effect on the Hippo pathway suppresses miRNA regulation among women; however, it should be noted that we do not have evidence to support such a mechanism.

This study has important limitations. One is the lack of measurement of the effects of differences in miRNA levels on messenger-RNA, protein synthesis, and cellular function. However, analysing messenger-RNA and proteomics data was outside the scope of this study. Furthermore, the study is limited by the fact that we only analysed a restricted set of miRNAs selected based on their association with AAA in men. This limits the ability to evaluate the deregulation of other miRNAs among women.

Aneurysm growth data were available only for participants with an aortic diameter > 25 mm (*n* = 110, including 43 women), and no strong associations were observed with growth rate, sex, or their interaction. The study was, however, underpowered to detect associations with aneurysm growth.

This study lacks data on aneurysm complexity, which would be expected to increase with larger aortic diameters. However, the observed reduction in the variance of miRNA levels with increasing aortic diameter suggests that the findings are unlikely to be explained by increasing complexity of aneurysmal disease.

The diagnostic criteria for AAA is a debated topic, especially when considering women. By using aortic diameter instead of disease categories, we have reduced the risk of misclassification.

Patients with known syndromic aneurysm diseases are not included in this cohort, although there is a very small risk that undiagnosed cases could have been included. These monogenetic syndromes are quite rare. If any of these undiagnosed patients were included, they would be single individuals, which would not affect the results.

We do not have exact data on menopause status, but since the youngest woman was 58 years old, we consider all of the women to be postmenopausal. There were no data on hormone replacement therapy (HRT), but as HRT was not recommended in Sweden beyond the age of 55 at the time of sampling, we consider it unlikely that any of the women were receiving HRT.

## 5. Conclusions

This exploratory pilot study found sex-dependent differences in the associations between miRNA levels and aortic diameter. The differences in AAA prognosis between men and women may be mirrored by differences in miRNA regulation. Describing the regulatory miRNA network in AAA disease provides new insights into AAA pathophysiology. Further studies on the biological implications of these sex differences in miRNA levels are warranted. Once again, it has been shown that the differences in the pathophysiology of AAA between men and women are greater than expected and need to be investigated further.

## Figures and Tables

**Table 1:** Participant characteristics.

Characteristic		Women	Men	*p*
		*n* = 85	*n* = 157	
		mean (95%CI)	mean (95%CI)	
Aortic diameter		29 (26–31)	25 (24–26)	0.370 ^1^
Age		72 (71–73)	71 (71–72)	0.110 ^1^
		% (95% CI)	% (95% CI)	
Coronary artery disease		20 (13–30)	15 (9.9–21)	0.277 ^2^
Hypertension		35 (26–46)	25 (19–32)	0.000 ^2^
Hypercholesterolaemia		8.2 (3.9–16)	3.8 (1.7–8.3)	0.072 ^2^
Cerebrovascular disease		13 (7.2–22)	12 (7.8–18)	0.537 ^2^
Claudication		39 (29–50)	32 (26–40)	0.142 ^2^
COPD		20 (13–30)	15 (9.9–21)	0.021 ^2^
Diabetes		35 (26–46)	25 (19–32)	0.838 ^2^
Renal failure		8.2 (3.9–16)	3.8 (1.7–8.3)	0.607 ^2^
Treatment—Statins		13 (7.2–22)	12 (7.8–18)	0.472 ^2^
Treatment—ASA		39 (29–50)	32 (26–40)	0.557 ^2^
Smoking status	Never	26 (18–36)	32 (26–40)	0.006 ^2^
	Previous	42 (32–53)	57 (49–64)	
	Active	26 (18–36)	11 (6.8–17)	

95%CI—95% confidence interval, ^1^ *p*-values calculated using Kruskal–Wallis, ^2^ *p*-values calculated using Fisher’s Exact Test.

**Table 2:** Multivariable regression estimating the association between miRNA levels and aortic diameter, sex, and their interaction.

	Aortic Diameter			Sex (Male)			Interaction Diameter: Sex		
miRNA	Estimate (95% CI)	*p*-Value	Adjusted *p*	Estimate (95% CI)	*p*-Value	Adjusted *p*	Estimate (95% CI)	*p*-Value	Adjusted *p*
10b	−0.061 (−0.3–0.17)	0.61	1	−0.15 (−0.49–0.19)	0.39	1	0.05 (−0.27–0.37)	0.76	1
124-3p	0.13 (−0.035–0.3)	0.12	1	−0.37 (−0.61–−0.13)	0.0031	0.052	−0.086 (−0.32–0.14)	0.46	1
125	0.092 (−0.12–0.3)	0.39	1	0.23 (−0.077–0.53)	0.14	1	−0.49 (−0.78–−0.21)	<0.001	0.013
128-1	−0.047 (−0.25–0.15)	0.64	1	−0.11 (−0.4–0.18)	0.45	1	−0.45 (−0.72–−0.18)	0.0013	0.017
145	−0.036 (−0.26–0.18)	0.75	1	0.092 (−0.23–0.41)	0.57	1	−0.37 (−0.67–−0.075)	0.014	0.13
194	−0.036 (−0.25–0.18)	0.74	1	0.26 (−0.041–0.57)	0.089	1	−0.49 (−0.78–−0.21)	<0.001	0.013
21	−0.065 (−0.27–0.14)	0.54	1	−0.17 (−0.47–0.13)	0.26	1	−0.36 (−0.65–−0.082)	0.012	0.12
24	−0.059 (−0.26–0.15)	0.57	1	0.023 (−0.27–0.32)	0.88	1	−0.48 (−0.76–−0.2)	<0.001	0.013
26a	−0.0056 (−0.22–0.21)	0.96	1	0.15 (−0.16–0.46)	0.34	1	−0.47 (−0.76–−0.18)	0.0018	0.022
326	−0.15 (−0.36–0.065)	0.17	1	−0.14 (−0.45–0.16)	0.35	1	−0.18 (−0.46–0.11)	0.22	1
335	0.061 (−0.15–0.27)	0.56	1	0.074 (−0.23–0.37)	0.63	1	−0.4 (−0.68–−0.12)	0.0057	0.063
451	0.0047 (−0.2–0.21)	0.96	1	0.27 (−0.028–0.57)	0.075	1	−0.21 (−0.49–0.075)	0.15	0.9
5706	−0.2 (−0.38–−0.0088)	0.04	0.68	−0.19 (−0.45–0.082)	0.17	1	0.095 (−0.16–0.35)	0.46	1
657	−0.15 (−0.36–0.061)	0.16	1	−0.011 (−0.32–0.29)	0.94	1	0.11 (−0.18–0.4)	0.46	1
671	0.04 (−0.17–0.25)	0.71	1	0.047 (−0.26–0.35)	0.76	1	−0.23 (−0.52–0.062)	0.12	0.86
93	−0.042 (−0.24–0.15)	0.67	1	0.066 (−0.21–0.35)	0.64	1	−0.54 (−0.8–−0.27)	<0.001	0.0015
let-7i	−0.16 (−0.38–0.052)	0.14	1	−0.0063 (−0.31–0.3)	0.97	1	−0.32 (−0.61–−0.027)	0.032	0.26

**Table 3:** Associated signalling pathways according to DIANA-mirPath.

KEGG Pathway (KEGG Identifier)	*p*-Value	Associated Gene Targets (N)	Associated miRNAs (N)
Hippo signalling pathway (hsa04390)	6.6 × 10^−13^	68	5
Proteoglycans in cancer (hsa05205)	2.7 × 10^−10^	92	6
TGF-beta signalling pathway (hsa04350)	1.3 × 10^−9^	42	5

Results from DIANA-mirPath v3, using empirical distributions. miRNA–gene associations are from Tarbase, except for miR-128-1, for which microT-CDS associations were used.

## Data Availability

The authors are open to share data with other researchers. Please contact the corresponding author with such requests.

## Supplementary Materials

The following supporting information can be downloaded at https://www.mdpi.com/article/10.3390/biomedicines14030507/s1. Table S1: Complete table of regression estimates.

## Abbreviations

The following abbreviations are used in this manuscript:

AAA	Abdominal aortic aneurysm
RNA	Ribonucleic acid
miRNA	MicroRNA
TGF-beta	Transforming growth factor beta
